# The Role of Outer Membrane Proteins and Lipopolysaccharides for the Sensitivity of *Escherichia coli* to Antimicrobial Peptides

**DOI:** 10.3389/fmicb.2018.02153

**Published:** 2018-09-07

**Authors:** Anna Ebbensgaard, Hanne Mordhorst, Frank M. Aarestrup, Egon B. Hansen

**Affiliations:** ^1^National Food Institute, Technical University of Denmark, Kongens Lyngby, Denmark; ^2^Department of Biology, University of Copenhagen, Copenhagen, Denmark

**Keywords:** antimicrobial peptides, Cap18, Lpp, OmpT, lipopolysaccharide

## Abstract

Bacterial resistance to classical antibiotics is emerging worldwide. The number of infections caused by multidrug resistant bacteria is increasing and becoming a serious threat for human health globally. In particular, Gram-negative pathogens including multidrug resistant *Escherichia coli* are of serious concern being resistant to the currently available antibiotics. All Gram-negative bacteria are enclosed by an outer membrane which acts as an additional protection barrier preventing the entry of toxic compounds including antibiotics and antimicrobial peptides (AMPs). In this study we report that the outer membrane component lipopolysaccharide (LPS) plays a crucial role for the antimicrobial susceptibility of *E. coli* BW25113 against the cationic AMPs Cap18, Cap11, Cap11-1-18m^2^, melittin, indolicidin, cecropin P1, cecropin B, and the polypeptide antibiotic colistin, whereas the outer membrane protease OmpT and the lipoprotein Lpp only play a minor role for the susceptibility against cationic AMPs. Increased susceptibility toward cationic AMPs was found for LPS deficient mutants of *E. coli* BW25113 harboring deletions in any of the genes required for the inner part of core-oligosaccharide of the LPS, *waaC, waaE, waaF, waaG*, and *gmhA*. In addition, our study demonstrates that the antimicrobial activity of Cap18, Cap11, Cap11-1-18m^2^, cecropin B, and cecropin P1 is not only dependent on the inner part of the core oligosaccharide, but also on the outer part and its sugar composition. Finally, we demonstrated that the antimicrobial activity of selected Cap18 derivatives harboring amino acid substitutions in the hydrophobic interface, are non-active against wild-type *E. coli* ATCC29522. By deleting *waaC, waaE, waaF*, or *waaG* the antimicrobial activity of the non-active derivatives can be partially or fully restored, suggesting a very close interplay between the LPS core oligosaccharide and the specific Cap18 derivative. Summarizing, this study implicates that the nature of the outer membrane component LPS has a big impact on the antimicrobial activity of cationic AMPs against *E. coli*. In particular, the inner as well as the outer part of the core oligosaccharide are important elements determining the antimicrobial susceptibility of *E. coli* against cationic AMPs.

## Introduction

The rapid emergence of bacterial resistance to classical antibiotics is occurring worldwide. The number of infections which are caused by multidrug resistant bacteria is increasing and bacterial infections have again become a threat. It's estimated that bacterial infections caused by multidrug resistant bacteria result in 25,000 deaths each year in Europe. In the USA, more than 2 million people are infected with antibiotic-resistant bacteria per year and 23,000 people are dying as a direct cause (Davies et al., 2013; World Health Organization, 2014). In particular, infections caused by Gram-negative bacteria which are resistant to almost all currently available drugs are of special concern. Multi-drug resistant Gram-negative bacteria are a great health threat due to limited treatment options. The pipeline for the development of new, efficient antimicrobials specifically targeting Gram-negative bacteria stays empty, despite the increasing number of multidrug resistant bacteria (Hampton, 2013). In particular, multidrug resistance in Enterobacteriaceae such as extended-spectrum β-lactamase (ESBL) and carbapenemase-producing *Escherichia coli* are of serious concern being resistant to the currently available antibiotics (Kanj and Kanafani, 2011; Antibiotics Currently in Clinical Development, 2014).

**Figure 1:**
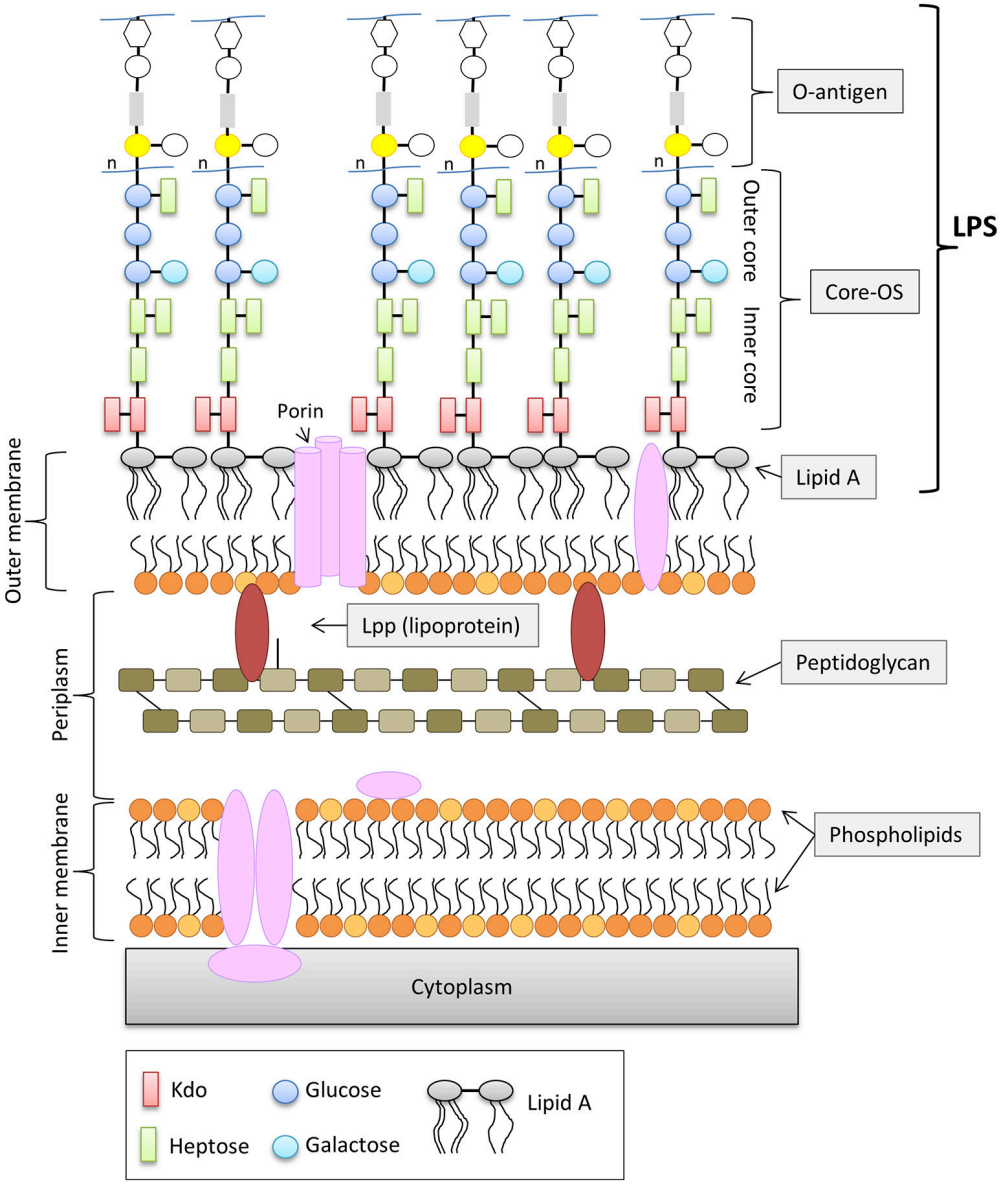
General structure of the cell envelope of *Escherichia coli* K-12. Schematic view of the cell envelope of *E. coli* K-12.

**Figure 2:**
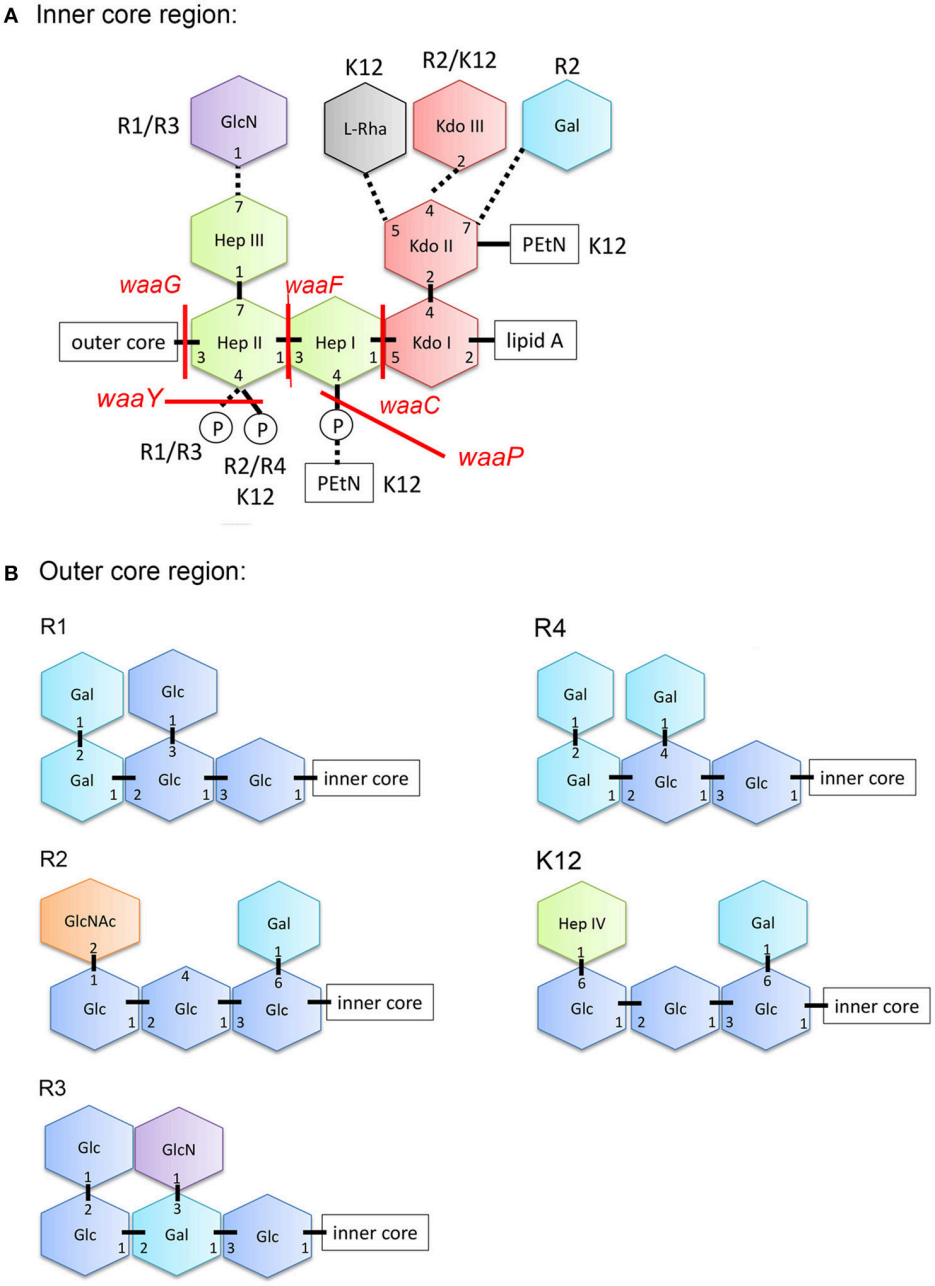
Structure of the core-OS of *Escherichia coli*. **(A)** Structure of the inner region of the core-OS of the *E. coli* core types. Dashed lines indicate non-stoichiometric substitutions and the distinguishing modifications of different core types are indicated. **(B)** Structure of the outer region of the core-OS of the R1, R2, R3, R4, and K-12 core types. Glc, glucose; GlcN, glucosamine; Hep, L-*glycero*-D-*manno*-heptopyranose; Kdo, 3-deoxy-D-*manno-*oct-2-ulosonic acid; Rha, rhamonse; Gal, galactose; GlcNAc, *N-*acetylglucosamine; PEtN, phosphorylethanolamine.

In contrast to Gram-positive bacteria, all Gram-negative bacteria are surrounded by an additional membrane, the so-called outer membrane (OM) (Figure 1). Unlike the cytoplasmic membrane, the OM is highly asymmetric, with an inner leaflet containing phospholipids, and an outer leaflet mainly composed of lipopolysaccharides (LPS). LPS of Gram-negative bacteria, including *E. coli* consists of three parts (Figure 1): (i) lipid A, the hydrophobic membrane anchor forming the outer leaflet of the OM, (ii) a phosphorylated, non-repetitive core oligosaccharide (core-OS) and therefore of highly anionic nature, and (iii) a O-antigen (O polysaccharide) made of a chain of several types of repeating sugar units acting as a hydrophilic coating surface (Erridge et al., 2002; Raetz and Whitfield, 2002). Lipid A is highly conserved among Gram-negative bacteria, in contrast to the O-antigen, which is highly flexible. The core-OS of *E. coli* is divided into an inner region composed of 2-keto-3-deoxy-D-*manno*-octulosonic acid (Kdo) and one or more L-*glycero*-D-*manno*-heptose residues and the outer region made of various linked sugar residues. The principal features of the inner region of the core-OS are conserved among members of the *Enterobacteriaceae*, which presumably reflects it's critical role in OM stability (Caroff and Karibian, 2003). In *E. coli*, the inner core region of the core-OS can be further modified with variable non-stochiometric substitution depending on the core-OS type (Heinrichs et al., 1998b). The outer region of the core-OS shows more variability which defines the five different core types in *E. coli* (K12, R1, R2, R3, and R4) (Figure 2). All core types share the structural theme with a (hexose)_3_ carbohydrate backbone and two side chain residues. However, the order of the hexose residues and the position, nature and linkage of the side chain varies from each core type (Figure 2). Most clinical isolates of *E. coli* have a smooth LPS (S-LPS) which consists of all three parts including the O-antigen, whereas *E. coli* with rough LPS (R-LPS) lacks the O-antigen and can even have a truncated core-OS (deep rough). The minimal LPS essential for growth consists of the Lipid A and Kdo (3-deoxy-D-*manno*-oct-2-ulosonic acid) (Frirdich and Whitfield, 2005).

The OM is essential for cell viability and serves as permeability barrier preventing the entry of harmful toxic compounds (Savage, 2001; Raetz and Whitfield, 2002; Nikaido, 2003). The overall composition and structure of the OM are widely conserved with only little variation among different species (Vaara, 1992). LPS—being the major component of the outer leaflet of the OM—plays a central role for the integrity and the selective permeability of the OM. Charged macromolecules are unable to penetrate the OM, while many hydrophobic molecules are allowed limited diffusion (Nikaido, 2003). LPS, in particular the core-OS plays an important factor in providing selectivity to hydrophobic molecules. Due to the anionic phosphate groups in the inner region of the core-OS, LPS molecules are able to form intermolecular electrostatic bonds with neighboring LPS molecules via divalent cations, mainly Mg^2+^ and Ca^2+^(Vaara, 1992). This cross-bridging of neighboring LPS molecules significantly contributes to the resistance against hydrophobic antimicrobial agents. The anionic nature of lipid A and the inner region of the core-OS seem to be the Achilles heel for the OM integrity (Schneck et al., 2010).

However, the OM of *E. coli* consists not only of LPS, but also of other components such as outer membrane proteins and lipoproteins (Figure 1). Lpp or murein-lipoprotein is the most abundant lipoprotein of *E. coli* and it is estimated to be numerically the most abundant protein with more than 500,000 molecules per cell (Vaara, 1992; Neidhardt and Umbarger, 1996). Lpp consists of 58 amino and exists in two forms, (i) the “bound form”, in which Lpp is covalently bound to the peptidoglycan layer via the ε-amino group of the C-terminal lysine (Nikaido, 1996) and (ii) the “free form” (Braun and Rehn, 1969; Braun and Wolff, 1970; Braun and Bosch, 1972). The function of the free from is not understood, however it has been shown that the “free from” is exposed to the surface by its C-terminus (Cowles et al., 2011). The “bound form” of Lpp is an important structural component in maintaining the stability and integrity of the OM. Cells lacking Lpp show several OM defects such as increased OM permeability to small, toxic molecules and antibiotics and leakage of periplasmic contents (Hirota et al., 1977; Suzuki et al., 1978).

Antimicrobial peptides (AMPs) are recapturing attention as a potential alternative to classical antibiotics fighting bacterial infections, including infections caused by Gram-negative pathogens. Cationic antimicrobial peptides represent the biggest class of AMPs and are present in virtually all groups of organisms such as bacteria, fungi, plants, and animals. Cationic AMPs are characterized by a positive charge and a hydrophobic region, but their sizes, sequences and secondary structures vary widely. It is widely believed, that the positive charge of cationic AMPs enables electrostatic interaction to the negatively charged microbial membranes (Yeaman and Yount, 2003; Jenssen et al., 2006) and that the hydrophobic region is involved in the penetration of the cells (Aoki and Ueda, 2013).

The aim of our work is to investigate the antimicrobial activity of selected cationic AMPs against Gram-negative bacteria, in particular *E. coli*. In particular we have studied the potential role of the outer membrane which performs a crucial role of providing an extra layer of protection acting as a selective barrier. More specifically, we analyzed the potential role of particular components of the OM such as LPS, Lpp, and OmpT, an outer membrane protease, in the antimicrobial activity of cationic AMPs. We measured the minimal inhibitory concentration of the cationic AMPs Cap18, Cap11, Cap11-1-18m^2^, melittin, indolicidin, cecropin P1, and cecropin B against *E. coli* BW25113 (K12) and isogenic mutants defective either in LPS components, Lpp, or OmpT. Further, we analyzed the susceptibility of *E. coli* F470, *E. coli* F632, *E. coli* F653, and *E. coli* F2513, representing the R1, R2, R3, or R4 prototypes, against the selected cationic AMPs. Finally, we investigated the mode of action of Cap18 by analyzing the antimicrobial activity of selected Cap18 derivatives against wild-type *E. coli* ATCC25922 as well as LPS mutants.

## Materials and methods

### Bacterial strains and growth conditions

All strains used in this study are listed in Table 1. The *E. coli* strains 470, F632, F653 and F2513 were kindly provided by Prof. H. Brade and Prof. S. Muller-Loennies, Research Center Borstel, Leipniz-Center for Medicine and Biosciences, Germany. *E. coli* BW25113 (CGSC#:7636), a derivative of F^−^, λ^−^ *E. coli* K12 strain BD792 is the wild-type strain which was used in the generation of the KEIO collection and ordered from the Coli Genetic Stock Centre (CGSC, Yale University). *E. coli* JW3596, *E. coli* JW3024, *E. coli* JW3595, *E. coli* JW3606, *E. coli* JW0212, *E. coli* JW1667, *E. coli* JW0554, *E. coli* JW3605 and *E. coli* JW3600 are isogenic mutants of *waaC, waaE, waaF, waaG, gmhA (lpcA), lpp, ompT, waaP*, or *waaY* derived from the parental strain *E. coli* BW25113 and were ordered from the KEIO collection (Baba et al., 2006). *E. coli* ATCC25922 is a clinical isolate, serotype O6, often used as control strain in antimicrobial susceptibility testing was ordered from the ATCC strain collection. All strains were grown in Mueller-Hinton-II medium and incubation took place aerobically at 37°C for 18–20 h.

**Table 1:** Bacterial strains used in this study.

**Strain**	**Relevant characteristics/genotype**	**References**
*E. coli* F470	*E. coli* R1 prototype; R-LPS derivative of O8:K27	Schmidt et al., 1969; Vinogradov et al., 1999
*E. coli* F632	*E. coli* R2 prototype; R-LPS derivative of O100:K? (B):H2	Hämmerling et al., 1971; Heinrichs et al., 1998a
*E. coli* F653	*E. coli* R3 prototype; R-LPS derivative of O100	Schmidt et al., 1970
*E. coli* F2513	*E. coli* R4 prototype; R-LPS derivative of O14:K7	Schmidt et al., 1974
*E. coli* BW25113	F-, Δ(araD-araB)567, ΔlacZ4787(::rrnB-3), λ-, rph-1, Δ(rhaD-rhaB)568, hsdR514 wild-type strain used in the KEIO collection	Datsenko and Wanner, 2000; Baba et al., 2006
*E. coli* JW3596	*E. coli* BW25113 *waaC::kan*	Baba et al., 2006
*E. coli* JW3024	*E. coli* BW25113 *waaE::kan*	Baba et al., 2006
*E. coli* JW3595	*E. coli* BW25113 *waaF::kan*	Baba et al., 2006
*E. coli* JW3606	*E. coli* BW25113 *waaG::kan*	Baba et al., 2006
*E. coli* JW0212	*E. coli* BW25113 *gmhA::kan* (*lpcA::kan*)	Baba et al., 2006
*E. coli* JW1667	*E. coli* BW25113 *lpp::kan*	Baba et al., 2006
*E. coli* JW0554	*E. coli* BW25113 *ompT::kan*	Baba et al., 2006
*E. coli* JW3605	*E. coli* BW25113 *waaP::kan*	Baba et al., 2006
*E. coli* JW3600	*E. coli* BW25113 *waaY::kan*	Baba et al., 2006
*E. coli* ATCC25922	Clinical isolate, Serotype O6, Biotype 1, control strain for antimicrobial susceptibility testing	ATCC strain collection
*E. coli* AD120	*E. coli* ATCC25922 Δ*waaC*	Ebbensgaard et al., 2015
*E. coli* AD121	*E. coli* ATCC25922 Δ*waaE*	Ebbensgaard et al., 2015
*E. coli* AD122	*E. coli* ATCC25922 Δ*waaF*	Ebbensgaard et al., 2015
*E. coli* AD123	*E. coli* ATCC25922 Δ*waaG*	Ebbensgaard et al., 2015

### Antimicrobial peptides

The amino acid sequence, origin and structure of cecropin P1, cecropin B, Cap18, Cap11, Cap11-1-12m^2^, melittin, and indolicidin are summarized in Table 2. Cecropin B, cecropin P1 and colistin sulfate (C4461) were purchased from Sigma-Aldrich. Cecropin B with a purity of ≥97% and cecropin P1 with a purity of ≥95% and were dissolved in water. Colistin sulfate was dissolved in water. Cap11 (94.7% purity), Cap11-1-18m^2^ (87.9% purity) and Cap18 (89.5% purity) were synthesized at Genscript. Cap18 and Cap11 were dissolved in DMSO, Cap11-1-18m^2^ was dissolved in water. Melittin (RP10290) and indolicidin (RP11242-0.5) each with a purity of >95% were purchased from Genscript and dissolved in water. The Cap18 derivatives used in this study were purchased as chemically synthesized peptides with high purity from Genscript or Peptide 2.0. The amino acid sequence and purity of each Cap18 derivative is summarized in Table 6. All peptides were dissolved to a stock concentration of 10 mg/ml, except colistin sulfate which was dissolved to a stock concentration of 50 mg/ml and stored at −20°C.

**Table 2:** Cationic antimicrobial peptides used in this study: sequence, origin, and structure.

**Peptide**	**Sequence**	**Origin**	**Structure**
Cap18	GLRKRLRKFRNKIKEKLKKIGQKIQGLLPKLAPRTDY	Mammalian, rabbit, neutrophils	α-helical
Cap11	GLRKKFRKTRKRIQKLGRKIGKTGRKVWKAWREYGQIPYPCRI	Mammalian, guinea pig, neutrophils	α-helical
Cap11-1-18m^2^	KLRKLFRKLLKLIRKLLR	Truncated derivative of Cap11	
Cecropin P1	SWLSKTAKKLENSAKKRISEGIAIAIQGGPR	Mammalian, pig, small intestine	α-helical
Cecropin B	KWKVFKKIEKMGRNIRNGIVKAGPAIAVLGEAKALG-NH_2_	Insects, giant silk moth, pupae	α-helical
Melittin	GIGAVLKVLTTGLPALISWIKRKRQQ-NH_2_	Insects, honey bee	α-helical
Indolicidin	ILPWKWPWWPWR-NH_2_	Mammalian, bovine neutrophils	extended

### Antimicrobial susceptibility testing (MIC testing)

The minimum inhibitory concentrations (MICs) of the AMPs were measured in 96-well microtiter plates according the Clinical and Laboratory Standards Institute [CLSI, formerly National Committee for Clinical Laboratory Standards (NCCLS)] (Wikler et al., 2009). Liquid Mueller-Hinton-II medium containing increasing concentrations of AMPs is inoculated with a defined number of cells (~10^5^ CFUs/ml) in 96-well microtiter plates (polypropylene). Each microtiter plate includes a positive growth control and a negative control (sterile control). After incubation, the minimal inhibitory concentration (MIC) is determined by the lowest concentration showing no visible growth. All plates were incubated for 16–20 h.

## Results

### OmpT has no or minor influence on the antimicrobial activity of cationic AMPs in the *escherichia coli* K12 BW25113

OmpT, a member of the aspartyl proteases, is found in the outer member of *E. coli* and is responsible for the cleavage of peptides and proteins between two consecutive basic amino acid residues (Sugimura and Nishihara, 1988; Kramer et al., 2000, 2001). Previously, it was shown that an *E. coli* Δ*ompT* mutant is hypersusceptible to the AMP protamine (Stumpe et al., 1998), and that OmpT of enterohaemorrhagic *E. coli* (EHEC) cleaves LL-37, the human homolog of Cap18 (Thomassin et al., 2012). In order to assess the role of OmpT in the K12 *E. coli* BW25133, we investigated the antimicrobial activity of seven cationic AMPs from different classes and origins as well as the antibiotic colistin against the *ompT* mutant which was selected from the KEIO collection (Baba et al., 2006). The antimicrobial activity was measured as minimal inhibitory concentration (MIC) and summarized in Table 3. The activity of the cathelicidin Cap18, a homolog of LL-37 is not affected in the *ompT* mutant compared to the parental strain BW25113 (Table 3). Cap11, another member of the cathelicidin family, is slightly more active, up to factor 2, in the *ompT* mutant strain compared to the wild-type strain. However, the MIC of Cap11-1-18m^2^, a shorter derivative of Cap11 is unchanged in the *ompT* background. Similar to Cap11, the antimicrobial activity of cecropin B and cecropin P1 is increased up to factor 2. In contrast, the antimicrobial activity of indolicidin is not affected in and melittin is slightly less effective (up to factor 2) in an *ompT* mutant background. The antimicrobial activity of the polypeptide antibiotic colistin is unchanged in the *ompT* mutant. The solvent DMSO had no antimicrobial activity in the concentrations used in the MIC assays (data not shown). Based on these results, we can suggest that the outer membrane protease OmpT has no or only a minor impact on the antimicrobial activity of cationic AMPs.

**Table 3:** Susceptibility of *E. coli* BW25113 mutants deficient in outer membrane to cationic antimicrobial peptides.

	**Cap18**	**Cap11**	**Cap11-1-18 m^2^**	**Melittin**	**Indolicidin**	**Cecropin P1**	**Cecropin B**	**Colistin**
*E. coli* BW25113	4–8	8	8–16	16–32	32	16–32	16–32	0.125–0.25
*E. coli* BW25113 *waaC::kan*	4	8	8	4	16	8	8	0.063–0.125
*E. coli* BW25113 *waaE::kan*	4	8	8	4	16	8	8	0.063–0.125
*E. coli* BW25113 *waaF::kan*	4	8	8	4	16	4	4	0.063
*E. coli* BW25113 *waaG::kan*	4	8	8	4	16	16	16	0.063–0.125
*E. coli* BW25113 *gmhA::kan*	4–8	8*	8*	2*	16*	4–8*	4–8*	0.063
*E. coli* BW25113 *ompT::kan*	4–8	4–8*	8*	32*	32*	16*	16*	0.125–0.25
*E. coli* BW25113 *lpp::kan*	2–4*	8*	8–16*	n.d.	n.d.	n.d.	16*	0.063–0.125
*E. coli* BW25113 *waaP::kan*	4–8	8–16	n.d.	4	32	16–32	32	0.125–0.25
*E. coli* BW25113 *waaY::kan*	4–8	8–16	n.d.	16–32	32	32	32–64	0.125–0.25

Data are collected as minimal inhibitory concentrations (MICs) according to the Clinical and Laboratory Standards Institute (CLSI) and expressed in μg/ml. All MIC determinations were carried out in duplicates

* *or in triplicates*.

*n.d., not determined*.

### Susceptibility testing of *E. coli* BW25113 *lpp::kan* to cationic antimicrobial peptides

Although it is known that the deletion of the *lpp* gene leads to increased drug susceptibility, the antimicrobial activity pattern of cationic AMPs has not been analyzed in a more detailed manner using isogenic strains. To investigate the potential role of Lpp on the antimicrobial activity of cationic AMPs, the BW25133 *lpp::kan* mutant was selected from the KEIO collection and tested for antimicrobial susceptibility. Due to the limited amounts of available peptide, only Cap18, Cap11, Cap11-1-12m^2^, cecropin B and colistin were tested for antimicrobial activity in the *lpp* mutant (Table 3). The MIC values for cecropin B, Cap11 and Cap11-1-12m^2^ were unchanged in the *lpp* mutant strain compared to the wild-type. Only, for Cap18 and colistin, a minimal increase of factor 2 in the antimicrobial susceptibility could be detected. Based on our results, we can conclude that Lpp is not playing a central role for the antimicrobial activity of the majority of the tested cationic AMPs. Only for Cap18 and colistin, Lpp plays a marginal role for the antimicrobial activity.

### Increase of antimicrobial susceptibility to cationic antimicrobial peptides in LPS deficient *E. coli* mutants

To understand the effect of LPS, in particular the core-OS, on the antimicrobial activity of cationic AMPs, several LPS mutants were selected from the KEIO collection and analyzed for antimicrobial susceptibility. Three different sets of LPS mutants were chosen, one set of mutants harboring deletions of genes involved in the assembly of the LPS molecule including glycosyl- and heptosyltransferases (*waaC, waaF*, and *waaG*), one set containing LPS modifying enzymes such as the LPS kinases *waaP* and *waaY*, and third set consisting of mutants deficient in the synthesis of ADP-heptose (*gmhA and waaE)* which is the precursor to the LPS heptose molecule. The MIC of Cap18, Cap11, Cap11-1-18m^2^, melittin, indolicidin, ceropin P1, cecropin B, and colistin was measured for *E. coli* BW25113 strains harboring a mutation either in *waaG, waaC, waaE, waaF*, or *gmhA*, all genes required for the core-OS of the LPS, and the wild-type strain BW25113. The MIC values are summarized in Table 3. Except for Cap11, all the tested cationic AMPs showed higher antimicrobial activity against the *waaC, waaF, waaG, waaE*, and *gmhA* mutants compared to the parental strain BW25113. The highest increase (8- to 16-fold) in antimicrobial activity was measured for melittin against BW25113 *gmhA::kan*. In addition, the susceptibility of BW25113 *waaC::kan*, BW25113 *waaF::kan*, BW25113 *waaG::kan* and BW25113 *waaE::kan*, was increased by a factor 4–8 compared to the wild-type strain. Cecropin P1 and cecropin B are more active in the LPS mutant backgrounds; 4–8x more active in a *waaF::kan* background, 4x more active in *gmhA::kan* background, 2–4x more active in *waaC::kan* and *waaE::kan* background and up to 2-fold more active in the *waaG::kan* background compared to the parental BW25113 strain. Indolicidin is twice as active and Cap11-1-18m^2^ shows a small increase of up to 2-fold in antimicrobial activity in all the LPS mutants tested compared to the wild-type strain. Similarly, deletions of *waaC, waaE, waaF* and *waaG* show a slightly increased susceptibility (up to 2-fold) against Cap18. In comparison, the polypeptide antibiotic colisitin exhibited a slightly increased antimicrobial activity (up to factor 2) against *waaC, waaF, waaG, waaE*, and *gmhA* mutants. In addition to the enzymes directly involved in the synthesis of the core oligosaccharide, we analyzed the effect of LPS modifying enzymes such as the LPS kinase *waaP* and *waaY* responsible for the phosphorylation of heptose I and heptose II. *WaaP* is phophorylating the heptose I residue, whereas *waaY* is responsible for the phosphorylation of the heptose II residue. However, in *waaP* mutant both sugar residues, heptose I and heptose II, remain unphosphorylated (Yethon et al., 1998). The antimicrobial susceptibility of a *waaP* mutant is not changed for Cap18, Cap11, cecropin B, cecropin P1, indolicidin and colistin. Only melittin is 4–8 times more active in a *waaP* mutant background compared to the wild-type strain. Inactivating *waaY* had no effect on the activity of Cap18, Cap11, cecropin P1, melittin, and indolicidin, whereas cecropin B was slightly less active (up to factor 2). Based on our results, we can conclude that LPS, in particular the sugar structure of the core-OS plays an important role for the antimicrobial activity of cationic AMPs. However, the gain of antimicrobial activity of tested AMPs against the individual LPS mutants is highly dependent on the nature of the AMP itself. The antimicrobial activity of Cap18, Cap11, and Cap11-1-18m^2^, all members of the cathelicidin family, is not or only marginal affected in all the tested LPS mutants compared to wild-type, whereas melittin is 4x more active in any of the tested LPS mutants with truncations in the sugar backbone. In contrast, the effect of phosphorylation of the heptose I and heptose II sugar residue is only minor. The antimicrobial activity of most of the tested AMPs as well as the polypeptide antibiotic colistin is unchanged in the *waaP* and *waaY*. Only melittin is 4 times more active in a *waaP* mutant, but not in a *waaY* mutant.

### The core type of *escherichia coli* determines the antimicrobial activity of cationic AMPs

To investigate the role of LPS in more detail, we analyzed the potential role of the different sugar moieties in the outer part of the core-OS in *E. coli*. Therefore, we determined the antimicrobial activity of the selected cationic AMPs against five *E. coli* strains representing the five different core types (R1, R2, R3, R4, and K12). The selected AMPs were tested for the antimicrobial activity against *E. coli* ATCC25922, *E. coli* BW25113 (K12), *E. coli* F470 (R1 prototype, rough LPS), *E. coli* F632 (R2 prototype, rough LPS), *E. coli* F653 (R3 prototype, rough LPS) and *E. coli* F2513 (R4 prototype, rough LPS). The antimicrobial activity was measured as minimal inhibitory concentration (MIC) and summarized in Table 4. The antimicrobial activity of Cap18 is very similar against all the tested *E. coli* strains with MIC values of 4–8 μg/ml. Only *E. coli* F2513 is slightly more susceptible to Cap18 with a MIC value of 2–4 μg/ml. The antimicrobial activity of Cap11, another member of the cathelicidin family, varies 2- to 4-fold depending on the core type. Cap11 shows highest antimicrobial activity against *E. coli* F2513, the R4 prototype. Similarly, Cap11-18m^2^, a short derivative of Cap11, is most potent against *E. coli* F2513. In contrast to the AMPs from the cathelicidin family, melittin shows the biggest variation in antimicrobial activity depending on the core type. The antimicrobial activity of melittin is e.g., 4- to 8-fold higher against *E. coli* F2513 (R4 core type) than BW25113 (K12). The MIC value of indolicidin against *E. coli* F2513 is 4- to 2-fold lower than the MIC values measured for the other strains. The MIC values measured for cecropin P1 are very similar for all strains measured indicating that the activity of cecropin P1 is independent on the core type. Similarly for cecropin B, a slight variation in MICs (≤2-fold) was measured depending on the core type. Summarizing, the antimicrobial efficacy of the tested cationic AMPs varies depending on the *E. coli* core type. All the tested cationic peptides are most active against *E. coli* F2513 which represents the R4 core type. This finding indicates that the sugar moieties of the outer core region of the core-OS might play an important role for the antimicrobial activity of cationic AMPs. However, the individual AMPs differs in their degree of LPS selectivity. Melittin showed the largest difference in MIC values ranging from 4 to 32 μg/ml depending on the isolate and core type. In contrast, the MIC values for Cap18 only show a minimal variation (up to factor 2) depending on the strain. This suggests that Cap18 targets *E. coli* in an efficient way regardless of the core type.

**Table 4:** Antimicrobial activity of selected antimicrobial peptides in *Escherichia coli* with different LPS core types.

**Antimicrobial activity** μ**g/ml**							
	**Cap18**	**Cap11**	**Cap11-1-18 m**^2^	**Melittin**	**Indolicidin**	**Cecropin P1**	**Cecropin B**
*E. coli* BW25113 (K12)	4–8	8	16–32	16–32	32	16–32	16–32
*E. coli* F470 (R1)	4	16	32	16	≥32	32	32
*E. coli* F632 (R2)	4	8	16–32	32	≥32	32	16–32
*E. coli* F653 (R3)	4–8	16	≥32	32	≥32	32	32
*E. coli* F2513 (R4)	2–4	4–8	8–16	4	8–16	16–32	16
*E. coli* ATCC25922	4	16	32	16	32	32	32

*Data are collected as minimal inhibitory concentrations (MICs) according to the Clinical and Laboratory Standards Institute (CLSI) and expressed in μg/ml. All MIC determinations were carried out in triplicates*.

### The antimicrobial activity of pattern of *E. coli* ATCC25922 is similar to core type R1

One of the most frequently used *E. coli* strains in antimicrobial susceptibility testing is *E. coli* ATCC25922. *E. coli* ATCC25922 is a clinical isolate with a smooth LPS (serotype 6), serves as a reference strain in antimicrobial susceptibility testing and was used in a previous study dissecting the antimicrobial activity of Cap18. The core type of ATCC25922 hasn't been identified so far. We measured the antimicrobial activity of the selected cationic AMPs against *E. coli* ATCC25922 and compared the MIC value to BW25311, F470, F632, F653, and F2513 all harboring a rough LPS and representing the different core types (Table 4). The antimicrobial susceptibility of ATCC25922 toward all the tested AMPs is very similar to F470. This indicates that ATCC25922 might be a member of the core type R1.

### Restoration of the antimicrobial activity of CAP18 derivatives by changing the structure of the inner core region of the LPS

In a previous study, we dissected the antimicrobial activity of Cap18 and were able to generate Cap18 derivatives with enhanced species-selective killing (Ebbensgaard et al., 2018). Together with our current findings demonstrating that the antimicrobial activity of Cap18 is not or only marginal affected by the presence of the inner core region or by the sugar composition of the outer region of the core-OS, we decided to investigate the mechanism of Cap18 and the role of LPS in more detail. For this analysis, we selected a set of Cap18 derivatives with species-specific killing properties. Based on our previous study, we selected 12 Cap18 derivatives which had lost their antimicrobial activity against *E. coli* ATCC25922 (MIC ≥ 64 μg/ml). Each of these 12 derivatives harbors one single amino acid substitution compared to the original Cap18 peptide. In addition, we selected 8 derivatives exhibiting the same antimicrobial activity as the original Cap18 peptide (MIC = 4–8 μg/ml) and were included in the study as control peptides. The amino acid sequences and purities of the selected Cap18 derivatives are summarized in Table 5. For all 20 Cap18 derivatives the antimicrobial activity was investigated measuring the MIC value against *E. coli* ATCC29522 Δ*waaC, E. coli* ATCC29522 Δ*waaE, E. coli* ATCC29522 Δ*waaF, E. coli* ATCC29522Δ *waaG* and the parental strain ATCC29522 (Table 6).

**Table 5:** Cap18 derivatives: amino acid sequence, purity, solvent, manufacturer.

**Cap18 peptide**	**Amino acid sequence**	**Purity**	**Solvent**	**Company**
Cap18-original	GLRKRLRKFRNKIKEKLKKIGQKIQGLLPKLAPRTDY	≥89.5%	DMSO	Genscript
L6P	GLRKR**P**RKFRNKIKEKLKKIGQKIQGLLPKLAPRTDY	99.9%	DMSO	Genscript
I13D	GLRKRLRKFRNK**D**KEKLKKIGQKIQGLLPKLAPRTDY	96.59%	DMSO	Peptide 2.0
I13P	GLRKRLRKFRNK**P**KEKLKKIGQKIQGLLPKLAPRTDY	98.67%	DMSO	Peptide 2.0
I13R	GLRKRLRKFRNK**R**KEKLKKIGQKIQGLLPKLAPRTDY	95.97%	DMSO	Peptide 2.0
I13F	GLRKRLRKFRNK**F**KEKLKKIGQKIQGLLPKLAPRTDY	100%	DMSO	Peptide 2.0
I13H	GLRKRLRKFRNK**H**KEKLKKIGQKIQGLLPKLAPRTDY	97.8%	DMSO	Genscript
I13M	GLRKRLRKFRNK**M**KEKLKKIGQKIQGLLPKLAPRTDY	100%	DMSO	Peptide 2.0
K16C	GLRKRLRKFRNKIKE**C**LKKIGQKIQGLLPKLAPRTDY	95.8%	DMSO	Genscript
K16D	GLRKRLRKFRNKIKE**D**LKKIGQKIQGLLPKLAPRTDY	99.84%	DMSO	Peptide 2.0
L17D	GLRKRLRKFRNKIKEK**D**KKIGQKIQGLLPKLAPRTDY	97.46%	DMSO	Peptide 2.0
L17K	GLRKRLRKFRNKIKEK**K**KKIGQKIQGLLPKLAPRTDY	96.84%	DMSO	Peptide 2.0
L17P	GLRKRLRKFRNKIKEK**P**KKIGQKIQGLLPKLAPRTDY	96.26%	DMSO	Peptide 2.0
K18P	GLRKRLRKFRNKIKEKL**P**KIGQKIQGLLPKLAPRTDY	99.32%	DMSO	Peptide 2.0
I20E	GLRKRLRKFRNKIKEKLKK**E**GQKIQGLLPKLAPRTDY	99.72%	DMSO	Peptide 2.0
I20N	GLRKRLRKFRNKIKEKLKK**N**GQKIQGLLPKLAPRTDY	96.95%	DMSO	Peptide 2.0
I24D	GLRKRLRKFRNKIKEKLKKIGQK**D**QGLLPKLAPRTDY	98.99%	DMSO	Peptide 2.0
I24G	GLRKRLRKFRNKIKEKLKKIGQK**G**QGLLPKLAPRTDY	96.2%	DMSO	Genscript
I24N	GLRKRLRKFRNKIKEKLKKIGQK**N**QGLLPKLAPRTDY	99.78%	DMSO	Peptide 2.0
G26T	GLRKRLRKFRNKIKEKLKKIGQKIQ**T**LLPKLAPRTDY	95.05%	DMSO	Peptide 2.0
L27P	GLRKRLRKFRNKIKEKLKKIGQKIQG**P**LPKLAPRTDY	97.94%	DMSO	Peptide 2.0

*Amino acid substitution is highlighted in bold*.

**Table 6:** Antimicrobial activity of Cap18 derivatives against *E. coli* ATCC25922 LPS mutants and other Enterobacteriaceae.

**Cap18 peptide**	**Antimicrobial activity: MIC** μ**g/ml**						
	***E. coli*** **ATCC25922**					***S*. typhimurium LT2**	***Y. ruckeri* 392/2003**
	**Wild-type**	**Δ*waaC***	**Δ*waaE***	**Δ*waaF***	**Δ*waaG***		
Cap18-original	4–8	2–4	4	2–4	4	4–8	2–4
L17K	≥64	8	8	8	8	16	≥64
I20E	≥64	4	4	4	8	8	32
I24G	≥64	8	8	8	8	8–16	32
L27P	≥64	8	4	4	4	8	32
I24N	≥64	8	16	8	16	32	≥64
I20N	≥64	16	16	8	16	8–16	≥64
I13R	≥64	32	≥64	16	8	16–32	32–64
I24D	≥64	16	16	16	≥64	≥64	≥64
I13P	≥64	32	32	16	32	16–32	≥64
I24D	≥64	16	16	16	≥64	≥64	≥64
I13D	≥64	≥64	≥64	≥64	≥64	≥64	≥64
L17D	≥64	≥64	≥64	≥64	≥64	≥64	≥64
L17P	≥64	≥64	≥64	≥64	≥64	≥64	≥64
I13F	4	2	2	2	2	4	2
I13H	4	2	2	2	2	4	8–16
K16D	4	2	8	2	4	4	4
L6P	8	4	8	4	4	8	64
I13M	8	4	8	4	4	8	4
K16C	8	4	8	4	8	8–16	8–16
K18P	8	2	4	4	4	4	32
G26T	8	4	4	4	4	4–8	2–4

*Data are collected as minimal inhibitory concentrations (MICs) according to the Clinical and Laboratory Standards Institute (CLSI) and expressed in μg/ml. All MIC determinations were carried out in in triplicates*.

The antimicrobial activity could be fully reconstituted for four derivatives by deleting any of *wwaC, waaE, waaF*, or *waaG* in the ATCC29522 background. In more detail, Cap18 harboring either a L17K, I20E, I24G or L27P substitution exhibit no antimicrobial activity against *E. coli* ATCC29522, but regained full antimicrobial activity (MIC value: 4–8 μg/ml) in LPS mutants harboring a deletion either in *wwaC, waaE, waaF*, or *waaG*. The Cap18 derivative with a I24N substitution regained full antimicrobial activity in a *waaC* and *waaF* mutant background compared to ATCC29522 wild-type. However, the deletion of *waaE* and *waaG* only led a partial restoration of the antimicrobial activity of I24N. Similarly, an I20N substitution could successfully restore the antimicrobial activity of Cap18 in a *waaF* mutant background and partially restore the activity in Δ*waaC*, Δ*waaE*, and Δ*waaF*. Cap18 I13R was not active in both the wild-type as well as in the *waaE* mutant (MIC≥64 μg/ml), however fully active in the *waaG* mutant and partially active in the *waaF* and *waaC* mutants. The antimicrobial activity of the Cap18 derivatives harboring a I13P or I24D substitution, both non-active in ATCC29522, could only be partially restored by deleting either *wwaC, waaE, waaF*, or *waaG*. Interestingly, Cap18 I13D, L17D and L17P were non-active against all the tested strains including the LPS mutants. Cap18 derivatives exhibiting an antimicrobial activity comparable to the original Cap18 peptide (MIC = 4–8 μg/ml), showed either unchanged or slightly improved (factor 2) antimicrobial activity in the LPS mutants compared to the original Cap18 peptide (Table 6). Summarizing, we could demonstrate that changing the LPS structure of *E. coli* ATCC25922, in particular the inner region of the core-OS, can lead to a restoration of the antimicrobial activity of individual Cap18 derivatives which were not active in the parental ATCC25922 strain.

## Discussion

Bacterial pathogens have evolved different mechanisms to resist the antimicrobial activity of cationic AMPs (Peschel and Sahl, 2006). The production of proteases degrading and thereby inactivating cationic AMPs is one obvious way for bacteria to protect themselves against antimicrobials. Several omptins such as OmpT, and PgtE, all proteases found in the OM of various Gram-negative pathogens belonging to the *Enterobacteriaceae* family, have been associated with the degradation of AMPs (Hritonenko and Stathopoulos, 2007). In particular, OmpT has been implicated in the proteolytic degradation of protamine and LL-37, the human homolog of Cap18. Measuring the MIC values for Cap18, melittin and indolicidin no change could be detected in an *ompT* mutant background compared the parental *E. coli* BW25113. Similarly, only a marginal increase in antimicrobial activity of Cap11, Cap11-1-18m^2^, cecropin P1 and cecropin B was detected in the *ompT* mutant strain compared to wild-type. Our data clearly indicate that OmpT only plays a minor role in protecting *E. coli* K-12 against cationic AMPs. This is in agreement with previous studies showing that the MIC of LL-37, the human homolog of Cap18, is unchanged in CFT073Δ*ompT* compared to the wild-type CFT073, an uropathogenic *E. coli* strain (Brannon et al., 2013). Similar results were observed in EPEC E2348/69, an enteropathogenic *E. coli*. The MIC value of LL-37 was unchanged in the Δ*ompT* mutant compared to the parental strain. For C18G, another AMP, a slight decrease in MIC (factor 2) was observed in the Δ*ompT* mutant (Thomassin et al., 2012). Even though there was no or only a minor effect of OmpT on the susceptibility, it has been demonstrated that OmpT from *E. coli* CFT073 and *E. coli* EPEC E2348/69 mediate degradation of LL-37(Thomassin et al., 2012; Brannon et al., 2013). The effect of OmpT on the susceptibility was more pronounced in the enterohemorrhagic *E. coli* EHEC EDL933. The MIC values for LL-37 was decreased by factor 2 and for C18G lowered by factor 8. Altogether, this indicates that the contribution of OmpT to the degradation and inactivation of AMPs is in general only marginal, however also depending on the AMP itself and the nature of the *E. coli* strain.

The nature of the cell surface, in particular the composition of the OM of Gram-negative bacteria has a major impact on the antimicrobial activity and efficacy of antimicrobial agents including cationic AMPs. Therefore, we investigated the role of Lpp, which is the most abundant protein in *E. coli* being present either as free surface exposed Lpp, or in bound form connecting the peptidoglycan with the OM. Recently, it was demonstrated that Lpp is binding specifically to the α-helical AMPs such as Cap18, LL-37 and SMAP-29, but not to Polymyxin B. A model has been suggested in which Lpp acts as receptor for Cap18, LL-37 and SMAP-29 contributing directly to the susceptibility of α-helical cationic AMPs (Chang et al., 2012). However, our data demonstrate that deleting the lpp gene leads to a slightly increased susceptibility which indicates that Cap18 efficiently kills *E. coli* BW25113 even in the absence of Lpp. Based on our findings, we conclude that the presence of Lpp is not required for the antimicrobial activity of the tested cationic AMPs such as Cap18, Cap11, Cap11-1-18m^2^ and cercopin B against *E. coli* BW25133. Depleting the cell for both forms of Lpp, the “free from” which is surface exposed, as well as the “bound form” with a clearly defined periplasmic function by deleting the *lpp* gene might result in a changed OM. Mutants deficient in total Lpp have been shown to produce OM vesicles and to leak periplasmic proteins (Hirota et al., 1977). More recently, the susceptibility pattern of various antibiotics has been investigated in L51, an *E. coli* K-12 derivative, and in the isogenic Δ*lpp* mutant. Similar to our results for cationic AMPs, the susceptibility was slightly increased for vancomycin, erythromycin and rifampicin in the Δ*lpp* mutant compared to the parental strain (Kowata et al., 2016). Even though it has been demonstrated that Lpp is able to bind α-helical cationic AMPs, Lpp seems to play only a marginal role for the antimicrobial susceptibility of *E. coli* to the cationic AMPs we tested in our study.

It is well-known that LPS plays a central role in membrane permeability and therefore a major impact on the antimicrobial activity of mainly hydrophobic antibiotics (Vaara, 1993; Delcour, 2009). One of the major interests of this study was to elucidate the role of LPS on the antimicrobial activity of cationic AMPs focusing on both, the impact of the structure of the inner and outer region of the core-OS as well as the phosphorylation of the core-OS. From our findings, we can conclude that changes of the structure of the inner region of the core-OS have a great impact on the antimicrobial activity of cationic AMPs and the majority of the tested AMPs exhibit increased antimicrobial activity in deep rough mutants of *E. coli* In *E. coli*, the inner core-OS region might have a protective function in preventing the binding of cationic AMPs to the inner core-OS, which is considered as the Achilles heel of LPS. Binding of cationic AMPs to the inner core-OS might cause enhanced lateral diffusion of the LPS. This is provoked by the displacement of divalent cations normally stabilizing neighboring LPS molecules by electrostatic interactions and leading to increased OM permeability. By deleting the outer region of the core-OS, the AMPs will get easier access to the cell membrane, which promotes the penetration of the AMP by self-promoted uptake. This is in agreement with previous studies which demonstrated that the outer region of the core-OS has a protective role against large cationic antimicrobial agents such as colicin N and that the outer region of the core OS weakens the non-specific interactions between colicin N and the membrane surface (Clifton et al., 2016). However, the degree of increased susceptibility highly depends on the nature of the AMP itself. The susceptibility of the tested mutants deficient in the inner core-OS region is e.g., unchanged for Cap11 compared to the wild-type strain, whereas the same mutants are 4–8 times more susceptible to melittin compared to the parental strain. Not only the nature of the AMP, but also the gene which has been deleted, seems to play a crucial role for the antimicrobial activity of some AMPs. Example, a *waaF* mutant exhibits a 4- to 8-fold increased susceptibility for cecropin P1 and cecropin B compared to wild-type, whereas a *waaG* mutant is only up to 2-fold more susceptible. The phosphorylation of the HepI and HepII sugar residue plays a minor role for the antimicrobial activity of the majority of tested AMPs, except for melittin. Only melittin is 4–8 more active in a *E. coli* lacking all the phosphate groups in the inner core region. However, it has been previously demonstrated that a *waaY* mutant, which is lacking the phosphate group on HepII, is less susceptible to LL-37, but not to other AMPs including CRAMP, SMAP-29, BMAP-27, Bac7, or polymyxin (Bociek et al., 2015). This findings highly suggests that *E. coli* with a phosphate-less inner OS-core region can be more or less susceptible to cationic AMPs suggesting more specific interactions of the individual AMPs and the LPS.

In addition, our findings suggest that not only the inner core region of the core-OS, but also the outer region of the core-OS is important for the mode of action of cationic AMPs in *E. coli*. The composition of the different sugar moieties of the outer core-OS region determines the antimicrobial activity of some of the tested cationic AMPs including Cap18, Cap11, Cap11-1-18m^2^, melittin, and indolicidin. *E. coli* F2513 representing the R4 core type is 4–8 time more susceptible for melittin than *E. coli* strains representing the K-12, R1, R2, or R3 core type. Similarly, indolicidin, Cap18, Cap11, and Cap11-1-18m^2^ are 2-fold more effective in *E. coli* F2513. In contrast, for cecropin B as well as for cecropin P1, the composition of the outer region of the core-OS has no influence on the antimicrobial activity. Taken together, these findings clearly indicate that the protective role of the outer core-OS region depends on the core type and the different sugar compositions. In order to identify the most potent AMP against one particular *E*. coli strain, analyzing the core type would be advantageous and useful prior to the administration of AMPs. Previously, a PCR based LPS core typing system has been developed for *E. coli* which allows the identification of the core type (Amor et al., 2000).

**Figure 3:**
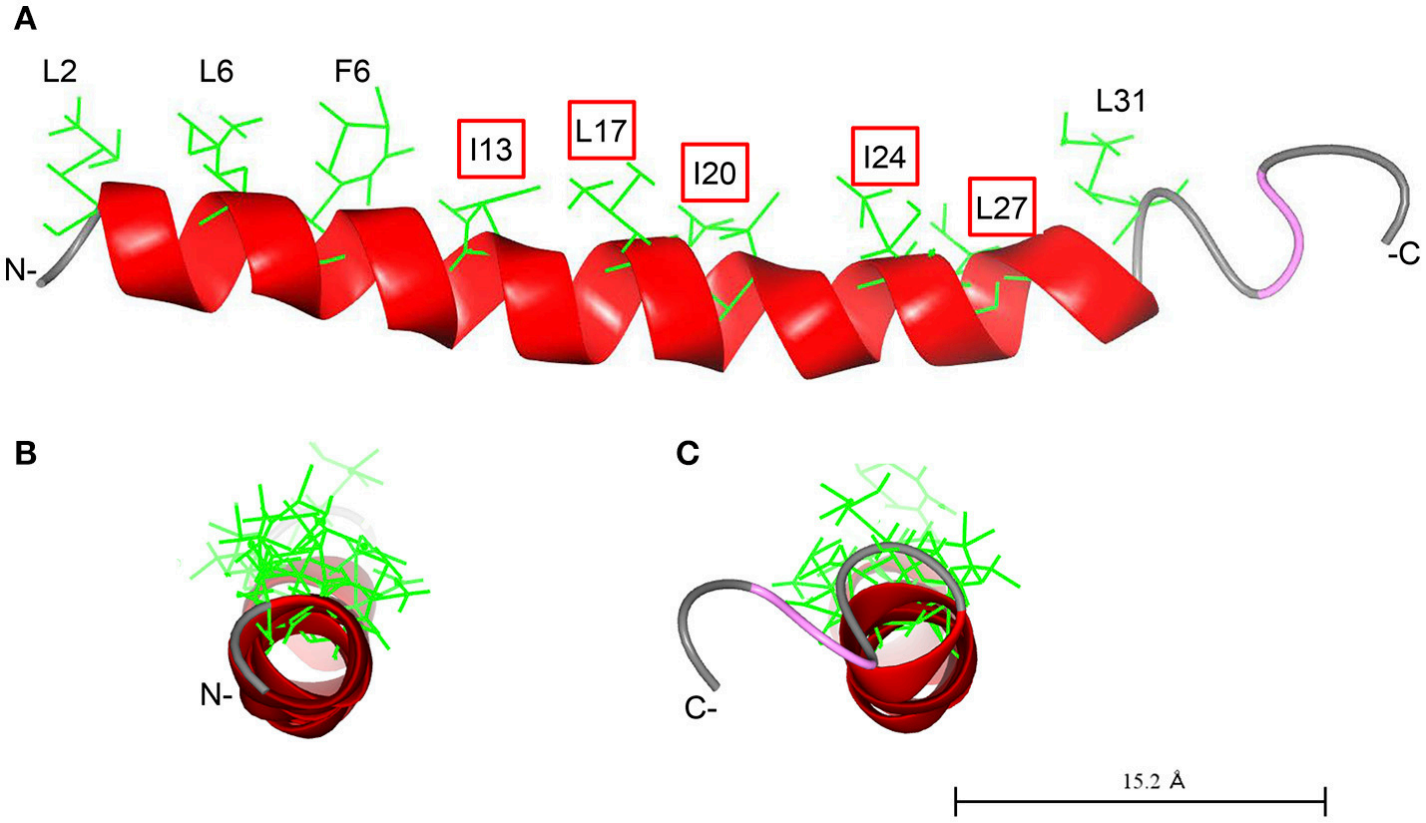
Predicted structure of Cap18. The structure of Cap18 was predicted using I-Tasser (Roy et al., 2010) and visualized by CCP4 software (McNicholas et al., 2011). The predicted α-helix is highlighted in red. Hydrophobic residues of the α-helix are shown in green. **(A)** View along helix axis, **(B)** view from N- to C-terminal, **(C)** view form C- to N-terminal.

As demonstrated previously, Cap18 is highly active against a broader range of bacterial pathogens including Gram-positive and Gram-negative bacteria and is a promising candidate for further applications (Ebbensgaard et al., 2015). In order to better understand the mode of action and interplay between Cap18 and the OM of *E. coli*, we investigated the antimicrobial activity of a selection of Cap18 derivatives in different *E. coli* mutant background with a changed inner core-OS region. Our data shows the importance of LPS for the antimicrobial activity of Cap18 and demonstrates that some of initially non-active Cap18 derivatives can regain full antimicrobial activity in specific LPS deficient backgrounds. Based on our results, we can conclude that an intact hydrophobic face of Cap18 plays a crucial role for the antimicrobial activity against *E. coli* ATCC25922, in particular the hydrophobic residues I13, L17, I20, I24, and L27 (Figure 3). The tested derivatives harboring a substitution in the hydrophobic face (I13, L17, I20, I24, L27) can be divided into two different groups. One group exhibits a complete loss of antimicrobial activity regardless of the LPS structure and a second group of Cap18 derivatives which were initially non-active against the wild-type, but regained full or partial antimicrobial activity in LPS mutants with a changed inner core-OS. Introducing the negatively charged residue aspartic acid either at position I13 or L17 destroys the antimicrobial activity of Cap18 against wild-type and LPS mutants regardless of the inner core-OS structure. This suggests that the changes of Cap18 provoked by the introduction of a negative charged residue at a central hydrophobic position have a dramatic effect on the antimicrobial activity which is in agreement with our previous study showing that Cap18 I13D and L17D lost activity against any of the tested organisms, regardless if Gram-positive or Gram-negative (Ebbensgaard et al., 2018). Similarly, the introduction of a proline residue at position L17 will lead to a complete loss of antimicrobial activity. In contrast, the antimicrobial activity of Cap18 I13R, in which a hydrophobic residue is substituted by the positively charged arginine, can be reestablished in deep rough mutants. Interestingly, the degree of antimicrobial activity of Cap18 I13R highly depends on the kind of LPS mutation, which suggests that the length of the LPS truncation determines the antimicrobial activity of Cap18 I13R. A LPS mutant only missing the outer region of the core-OS is the more susceptible than a heptoseless mutant only consisting of the minimal Kdo2-LipidA unit. Similarly, the antimicrobial activity of Cap18 L17K can be partially regained by changing the inner core-OS structure. Interestingly, the very same derivative was shown to exhibit species-specific antimicrobial activity only killing *P. aeruginosa*, but not targeting *Y. ruckeri, L. lactis*, and *E. faecalis* (Ebbensgaard et al.). These findings suggest that the antimicrobial activity of Cap18 L17K is dependent on the structure of the bacterial cell wall. Similarly, derivatives with either an I20E, I20N, I24G, I24N, or L27P substitution, initially inactive against wild-type *E. coli*, are regaining full or partial activity by changing the LPS structure. To summarize, we suggest a close interplay between LPS and Cap18 which is very specific for the tested Cap18 derivative and highly dependent on the structure of the inner core-OS.

## Author contributions

AE, EH, and FA have conceived the study, were in charge of the overall study design and took part in the project discussions. AE and HM have performed all experimental work. AE and EH composed the manuscript. AE, HM, FA, and EH contributed to manuscript revision and approved the submission.

### Conflict of interest statement

The authors declare that the research was conducted in the absence of any commercial or financial relationships that could be construed as a potential conflict of interest.
